# Variants of Insulin-Signaling Inhibitor Genes in Type 2 Diabetes and Related Metabolic Abnormalities

**DOI:** 10.1155/2013/376454

**Published:** 2013-05-23

**Authors:** Carlo de Lorenzo, Annalisa Greco, Teresa Vanessa Fiorentino, Gaia Chiara Mannino, Marta Letizia Hribal

**Affiliations:** Department of Medical and Surgical Sciences, University of Catanzaro “Magna Graecia”, 88100 Catanzaro, Italy

## Abstract

Insulin resistance has a central role in the pathogenesis of several metabolic diseases, including type 2 diabetes, obesity, glucose intolerance, metabolic syndrome, atherosclerosis, and cardiovascular diseases. Insulin resistance and related traits are likely to be caused by abnormalities in the genes encoding for proteins involved in the composite network of insulin-signaling; in this review we have focused our attention on genetic variants of insulin-signaling inhibitor molecules. These proteins interfere with different steps in insulin-signaling: ENPP1/PC-1 and the phosphatases PTP1B and PTPRF/LAR inhibit the insulin receptor activation; INPPL1/SHIP-2 hydrolyzes PI3-kinase products, hampering the phosphoinositide-mediated downstream signaling; and TRIB3 binds the serine-threonine kinase Akt, reducing its phosphorylation levels. While several variants have been described over the years for all these genes, solid evidence of an association with type 2 diabetes and related diseases seems to exist only for rs1044498 of the *ENPP1* gene and for rs2295490 of the *TRIB3* gene. However, overall the data recapitulated in this Review article may supply useful elements to interpret the results of novel, more technically advanced genetic studies; indeed it is becoming increasingly evident that genetic information on metabolic diseases should be interpreted taking into account the complex biological pathways underlying their pathogenesis.

## 1. Introduction

**Figure 1:**
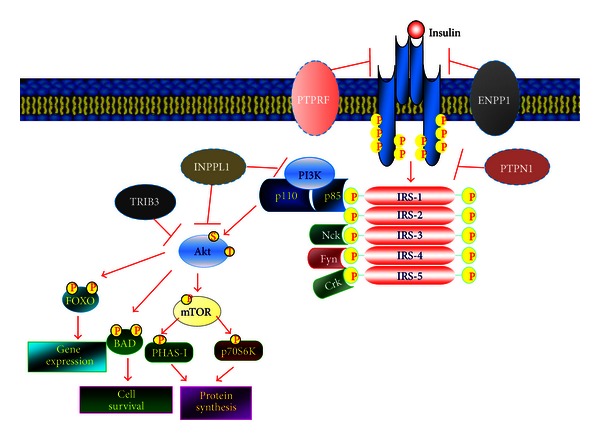
Schematic representation of the insulin-signaling pathway. Dashed light-blue line borders indicate insulin-signaling inhibitor proteins. PTPRF = protein tyrosine phosphatase receptor type F; ENPP1 = ectonucleotide pyrophosphatase/phosphodiesterase 1; PTPN1 = protein tyrosine phosphatase nonreceptor type 1; IRS = insulin receptor substrate; PI3K = phosphoinositides 3 kinase; nck = noncatalytic region of tyrosine kinase adaptor protein 1; INPPL1 = inositol polyphosphate phosphatase-like 1; TRIB3 = tribbles homolog 3; mTOR = mammalian target of rapamycin; Foxo = forkhead box protein O1; BAD = Bcl-2-associated death promoter; PHAS-I = phosphorylated heat-and acid-stable protein regulated by insulin; and p70S6K = p70-ribosomal S6 kinase.

Insulin is the primary anabolic hormone known and it regulates several processes, including cellular growth, differentiation, apoptosis, and lipid, protein, and glucose synthesis and breakdown [1]. The first step of insulin action involves its binding to the insulin receptor (IR) and the consequent activation of the receptor intrinsic tyrosine kinase activity. Once activated, the IR catalyzes phosphorylation of other proteins, such as the IR substrate proteins (IRS1, IRS2, IRS3, and IRS4), which, in turn, act as docking molecules for SH2-domain containing proteins, including the regulatory subunits of Phosphoinositides 3 kinase (PI3K). PI3K then catalyzes the phosphorylation of the 3′ hydroxyl subunit of phosphoinositides (PIs), notably converting PtdIns(4,5)P2 (PIP_2_) to PtdIns(3,4,5)P3 (PIP_3_), thus activating an assorted group of signaling proteins, containing phosphoinositide-binding domains. The activation of these proteins subsequently leads to the phosphorylation and activation of the serine-threonine kinase Akt (also known as protein kinase B) that ultimately transmits the insulin signal to a branching series of intracellular pathways that regulate cell differentiation, growth, survival, and metabolism [2]. Several molecules that inhibit this complex pathway at different levels have been described; among them: ectonucleotide pyrophosphatase/phosphodiesterase (ENPP1), the phosphatases protein tyrosine phosphatase nonreceptor type 1 (PTP1B), and protein tyrosine phosphatase receptor type F (PTPRF) inhibit the IR activation [3–5]; inositol polyphosphate phosphatase-like 1 (INPPL1) hydrolyzes PI3-kinase products, hampering the phosphoinositide-mediated downstream signaling [6]; and tribbles homolog 3 (TRIB3) binds Akt, reducing its phosphorylation levels [7] (Figure 1). An impaired activation of the insulin-signaling pathway results in a decreased responsiveness of target tissues to normal circulating levels of insulin, a condition known as insulin resistance. Insulin resistance has a central role in pathogenesis of several metabolic diseases, as it not only plays a major role in the development of type 2 diabetes mellitus (T2D) but is also a feature of a number of related disorders, including obesity, glucose intolerance, dyslipidemia, and hypertension, clustering in the so-called metabolic syndrome [2], atherosclerosis and cardiovascular diseases (CVD) [8]. Insulin resistance and related traits are likely to be caused by abnormalities in the genes encoding for proteins involved in the composite network of insulin-signaling; however, surprisingly, a very limited number of the loci identified by genome-wide (GWAS) studies as associated with T2D and related diseases seem to directly affect insulin action [9, 10]. Several hypothesis have been proposed to explain this unexpected fact and have been authoritatively reviewed elsewhere [9, 10]; here we will simply point out that the added effect of the variants identified so far explains less than 10% of T2D heritability, thus likely representing only the tip of the iceberg of the intricate genetic architecture of T2D. In this review, we will summarize the available data on variants of genes encoding for insulin-signaling inhibitor molecules and their association with insulin resistance and related diseases. To this end, we have performed a literature search using MEDLINE PubMed with different combinations of the following search terms: “ENPP1”, “NPP1”, “PC-1”, “TRIB3” “TRB3” “NIPK”, “LAR”, “PTPRF”, “R2A PTP”, “PTP1B”, “PTPN1”, “PTPN11”, “SHIP-2” “INPPL1”, “genetics of insulin resistance”, “genetics of type 2 diabetes”, “genetics of cardiovascular disease”, “genetics of metabolic syndrome”, “diabetes”, “variant”, “polymorphism”, and “genotype”.

## 2. ENPP1/PC-1

**Figure 2:**
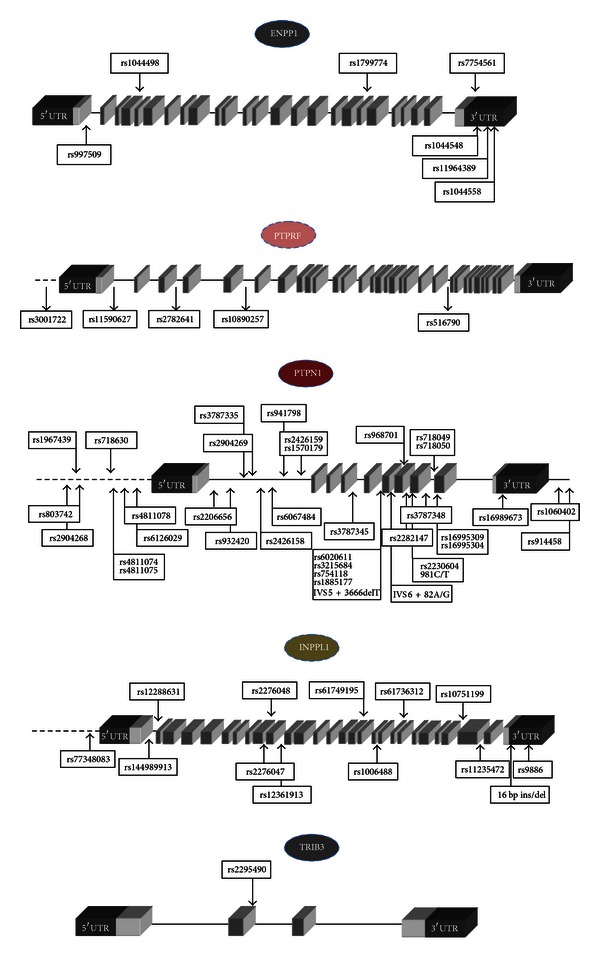
Genomic structure of insulin-signaling inhibitor molecules with selected single nucleotide polymorphisms (SNPs). Disease-associated SNPs are reported in boxes. Dark grey blocks = 5′ and 3′UTR regions; light gray blocks = exons; dotted lines-promoter regions.

ENPP1, also known as PC-1 (plasma cell-1), is a class II transmembrane glycoprotein that interacts with the IR and inhibits subsequent insulin-signaling by decreasing its beta-subunit autophosphorylation [3]. Transgenic animals that overexpress ENPP1 in different tissues are insulin resistant and diabetic [11]. Several variants of the *ENPP1/PC-1* gene have been described (Figure 2). The most widely investigated *ENPP1* variant is rs1044498A/C, a missense polymorphism, where a lysine, K, is substituted by a glutamine, Q, at codon 121 (or 173 depending on whether the downstream or the 156-bp upstream ATG triplet is considered as the start codon) [12]. From a molecular point of view, the Q121 SNP is a “gain of function” variant as the mutant ENPP1 shows *in vitro* an increased inhibitory activity [13, 14]. Transfection of the Q121 ENPP1 variant in HepG2 human hepatoma cells or in rat skeletal muscle L6 cells [14] resulted in a greater reduction of the IR autophosphorylation than transfecting the K121 form. Notably, this greater inhibitory effect on IR autophosphorylation was retained at downstream post receptor steps and resulted in a more profound inhibition of tissue-specific insulin action (glucose uptake and glycogen synthesis, resp.). Remarkably, data on either transfected INS-1E cells or isolated human islets suggest that Q121 ENPP1 also affects both function and survival of pancreatic beta cells [14]. These recent *in vitro* data are in keeping with those from an earlier case-control study showing that early-phase insulin secretion is significantly impaired in QQ individuals but not in heterozygous KQ subjects, from two Italian cohorts, comprising, respectively, 746 adult nondiabetic individuals and 289 obese/overweight children [15]. 

*In vivo*, the Q121 allele has been associated with quantitative traits related to insulin resistance in many but not all studies [16–26]. Some of these associations, however, were clearly driven by interaction with either specific subphenotypes [18, 23] or other genetic background features [19]. Several studies have also investigated whether the Q121 variant is more prevalent among patients with T2D than in the nondiabetic population, obtaining conflicting results [18, 22, 23, 25, 27–31]. However, a meta-analysis, including all studies published until 2008, suggested that European carriers of the QQ genotype are at increased risk of T2D (38% increased risk; *P* = 5 × 10^−3^), even if this association did not reach a genome-wide level of significance [32]. Indeed, several large, genome-wide association studies, either considered alone or when meta-analyzed, found no association between the rs7767502C/G variant (which is in perfect linkage disequilibrium with the K121Q polymorphism) and T2D [33]. These contradictions could be attributable to several factors, including the fact that Q121 shows a recessive model of risk transmission, which has not been tested so far in GWAS and the low frequency (approximately 3%) of the QQ genotype in the general population. In fact, with this frequency, a sample size of approximately 52,000 individuals would be needed to have a 90% power to detect a 38% increased risk for individuals with the QQ genotype at a genome-wide level of significance [9]. Furthermore, a few studies suggest that the K121Q polymorphism of *ENPP1/PC-1 *may have a stronger effect on the risk of early-onset T2D [27, 29]; thus more significant results may be obtained restricting the GWAS to early-onset cases [34]. Three studies have addressed also the hypothesis that rs1044498 may determine susceptibility to environmental changes and could thus predict the success of lifestyle intervention in treating T2D. In a first study, Moore and colleagues showed that while rs1044498 was associated with increased T2D incidence in 3584 subjects participating in the Diabetes Prevention Program (DPP) study, life style intervention abolished this increased risk [35]. In a subsequent study, the Q variant was, by contrast, not associated with increased risk for T2D in 1563 individuals with family history for the disease but was demonstrated to affect the change in insulin sensitivity during lifestyle intervention, with Q carriers showing an impaired increase in OGTT-derived insulin sensitivity [36]. Finally, in a recent study on a cohort of 211 overweight/obese nondiabetic subjects, Q allele carriers have been reported to be highly responsive to weight loss-induced improvement of fasting glucose levels [37]. These three studies suggest that rs1044498 has the potential to be implemented, in the next future, as a genetic marker for clinical use.

In addition to T2D, the *ENPP1/PC-1* Q121 allele has also been reported to influence the risk of obesity [29, 31, 38–40], a condition characterized by insulin resistance. There is also evidence suggesting that the Q121 allele is associated with proatherogenic phenotypes [41] and an increased risk of earlier onset of myocardial infarction (MI) [27, 42]. More recently Bacci et al. [43] reported that the K121Q polymorphism is an independent predictor of major cardiovascular events (MI, stroke, and cardiovascular death) in three cohorts of very high-risk individuals (patients with T2D and coronary artery disease (CAD)), patients with MI and without T2D and patients without T2D, and with end stage renal diseases. In type 2 diabetes, this effect was exacerbated by obesity.

In addition to rs1044498, other *ENPP1/PC-1* variants have been reported to modulate insulin resistance-related metabolic disturbances. In a large study [29], a three-polymorphism “risk haplotype” of the *ENPP1* gene has been described to be associated with obesity and T2D in both children and adults. This haplotype included the previously reported Q121 allele variant and two functionally uncharacterized noncoding polymorphisms: rs1799774-/T and rs7754561A/G, the latter being located in the 3′UTR, which might be involved in the modulation of gene expression. In subjects with this haplotype, ENPP1 levels in blood are elevated, suggesting that both enhanced expression and function are present. The same haplotype has also been subsequently reported to predict hyperglycemia in children from Germany [40] but not in adults of several different ethnicities [44]. Additional polymorphisms in the gene regulatory region (either in the 3′ or in the promoter region) have been suggested to be associated with T2D [28, 45]. Bochenski et al. [28] reported a significant association of rs997509 in intron 1 of the ENPP1 gene with T2D in a Polish cohort of obese subjects. In addition, Frittitta et al. [45] described a haplotype (a cluster of three single nucleotide polymorphisms: rs1044548G/A, rs11964389G/C, and rs1044558C/T) in the 3′-untranslated region of the *ENPP1* gene that may modulate ENPP1 expression and confer an increased risk for insulin resistance. Individuals from Sicily, Italy, carrying the “P” haplotype (rs1044548 A, rs11964389 C, and rs1044558 T) were at higher risk for insulin resistance and had higher levels of plasma glucose and insulin during an oral glucose tolerance test (OGTT) and higher levels of cholesterol, HDL cholesterol, and systolic blood pressure. Interestingly, the evaluation of ENPP1 protein content in skeletal muscle biopsies and cultured skin fibroblasts from a subset of the original cohort revealed that the P haplotype was also associated with increased ENPP1 expression [45].

## 3. PTPRF and R2A PTP Subfamily

PTPRF, also known as human leukocyte antigen related (LAR), belongs to the receptor type IIA (R2A) subfamily of protein tyrosine phosphatases (PTPs). The R2A PTP subfamily includes PTPRF, PTPR sigma (PTPRS), and PTPR delta (PTPRD), and it has been implicated in neural development, cancer, and diabetes [46]. PTPRF was demonstrated to be expressed in several insulin sensitive tissues where it interacts with insulin receptor and dephosphorylates its tyrosine-kinase domain [4]. PTPRF overexpression has been shown to induce insulin resistance in transgenic mice, and insulin receptor tyrosine phosphorylation and kinase activity were found to be increased by the reduction of PTPRF expression [47, 48]. Furthermore, PTPRF was overexpressed in adipose and skeletal muscle tissues of obese insulin-resistant human subjects [49, 50]. 

Miscio et al. analyzed the entire sequence of the *PTPRF* gene and identified two SNPs in the promoter region (a C to G change at −133 bp and a T to A change at −127 bp from the transcription start site) and a six-base insertion/deletion (GGCTCC) at +92 bp from the transcriptional start site in the first exon (Figure 2). Two of these variants were not further considered due to their low-allelic frequency (0.8% and 0.7% resp.), while the T to A change at −127 bp (rs3001722A/T) showed an allelic frequency of 5% and was tested for association with insulin resistance [51]. The analysis of 589 nondiabetic Caucasian residents of the Gargano area revealed that the allele A (minor allele) was significantly associated to lower body mass index (BMI), waist circumference, and mean blood pressure. The risk of having a high BMI value was reduced by approximately 60% in allele A carriers. In addition, allele A was associated to lower triglycerides, glucose, and insulin levels during an OGTT in an independent population including 307 individuals from East Sicily [51]. Functional studies, carried out in HEK293 human embryonic kidney cells, showed that the promoter activity of allele A was similar to that of allele T [51].

Subsequently, the association between *PTPRF* genetic variants and CAD in T2D patients has been evaluated in a study carried out in a cohort of 592 subjects enrolled at the Scientific Institute CSS-San Giovanni Rotondo, Italy. Four polymorphisms (rs11590627C/T, rs2782641A/G, rs10890257C/T, and rs516790G/T), tagging three linkage disequilibrium blocks, were genotyped (Figure 2). CAD was significantly associated with rs2782641, that resides within intron 3, and it was found to be in linkage disequilibrium with six additional SNPs (rs6695915A/G, rs651740C/T, rs2842187A/G, rs2819339G/T, rs2842185A/G, and rs11580074A/G). The association between rs2782641 and CAD was consistent with a recessive model of inheritance, with rs2782641 GG genotype carriers having a 50% increased risk of CAD in comparison to subjects with AA or AG genotype [52].

A second member of the R2A PTP subfamily, PTPRS, has been reported to be expressed in insulin target tissues, such as liver, adipose tissue, skeletal muscle, and endothelial cells [53]. PTPRS-deficient mice exhibit lower plasma glucose and insulin levels and greater insulin sensitivity than wild-type controls, suggesting that PTPRS may affect insulin action, even if it is unclear if it is able to directly dephosphorylate the IR or indirectly modulates its activation [54]. Notably the *PTPRS* gene is located on chromosome 19p13.3, a region that has been suggested to influence traits underlying lipid abnormalities associated with T2D. The association between *PTPRS* gene polymorphisms and T2D and impaired glucose tolerance (IGT) susceptibility has been evaluated in a study performed on 1057 Swedish Caucasians including 497 subjects with normal glucose tolerance (NGT), 262 with IGT, and 298 patients with T2D. A total of 8 SNPs were analyzed and three of them were reported to be associated with T2D. No association was found between *PTPRS* genetic variants and IGT. SNP rs1143699C/T located in exon 33 was associated with an increased risk of T2D in male patients while SNP rs4807015C/T and rs1978237C/G, located in intron 34 and 13, respectively, were associated with T2D risk in both genders [55].

More recently, a two-stage GWAS study performed on a Han Chinese population (2798 T2D patients and 2367 controls) identified rs17584499C/T as a novel locus associated with T2D susceptibility (*P* = 8.54 × 10^−10^; odds ratio [OR] = 1.57; 95% confidence interval [CI] = 1.36–1.82). rs17584499 lies within intron 10 of the gene encoding for an additional member of the R2A, subfamily, PTPRD [56]. Although no data on PTPRD role in insulin-signaling have been reported to date, as stated above R2A subfamily members are structurally very similar [46]; it is thus plausible to hypothesize that this protein may be involved in insulin action. The association observed in the GWAS study was subsequently replicated in a large family cohort study; Chang YC showed that, over an average follow-up period of 5.43 years, nondiabetic Han Chinese subjects carrying the rs17584499 TT genotype were more likely to develop diabetes in comparison to noncarriers. The risk-conferring T allele was associated with a greater increase in homoeostasis model assessment of insulin resistance (HOMA-IR) over time [57]. The association between rs17584499 and T2D in Han Chinese population was further confirmed in a recently published study carried out on 197 diabetic patients and 212 healthy controls. Interestingly, in this study rs17584499 was found to be associated also with pioglitazone therapeutic efficacy. In fact, patients with rs17584499 CT+TT genotypes showed significantly lower differential value of postprandial plasma glucose compared to those with CC genotype after pioglitazone treatment for 3 months [58].

## 4. PTPN1

The protein tyrosine phosphatase nonreceptor type 1 (*PTPN1*) gene encodes for the protein tyrosine phosphatase 1B (PTP1B), which suppresses the signaling pathway of insulin [5]. Several polymorphisms that colocalize with *PTPN1* have been analyzed in blocks, since the examination of the patterns of linkage disequilibrium in this region revealed very limited haplotype diversity within populations. The composition of the linkage blocks in different studies is mainly overlapping, with some exceptions due to the assumptions made by the authors, the study design, and, as mentioned, the distribution of the frequencies of the SNPs in the study populations. In this review, we will cover the scientific reports of both complex haplotypes and specific genetic variants (Figure 2). 

To begin with, the minor alleles of three tag SNPs of *PTPN1*—rs6067484A/G, rs6020611A/G, and rs1060402A/G—and the major allele at rs3787348G/T have been associated with higher levels of total plasma cholesterol and low-density lipoprotein (LDL) cholesterol in men with a BMI below 26 kg/m^2^ [59]. In a previous study, though, the same haplotype showed no effect on different measures of obesity, macronutrient intake, or eating behavior [60]. Bento et al. [61] performed a genetic analysis on rs3787348G/T together with other 22 SNPs (rs2904268C/G, rs803742C/T, rs1967439A/G, rs718630A/C, rs4811078C/T, rs2206656C/G, rs932420C/T, rs3787335G/T, rs2426158A/G, rs2904269A/C, rs941798A/G, rs1570179C/T, rs3787345C/T, rs1885177A/C, rs754118T/C, rs3215684ins-/T, rs968701C/T, rs2282147A/G, rs718049C/T, rs718050A/G, rs16989673ins-/G, and rs914458C/G) spanning the 161 kb region encoding *PTPN1* and the 5′ and 3′ UTR. Thus, they demonstrated the association of *PTPN1* polymorphisms with T2D in two independently ascertained Caucasian case-control populations, with overall odds ratios of ~1.3 [61]. The same group evaluated these SNPs and haplotypes for association with quantitative glycemic traits in a third independent sample, confirming that the protective haplotype led to higher insulin sensitivity and lower fasting glucose [62]. In the attempt to assess the effects of the common variants of *PTPN1* on measures of adiposity, insulin resistance, and metabolic syndrome, the above mentioned set of SNPs was screened in a large sample of healthy Caucasian female twins [63]. SNP rs718049C/T was significantly associated with waist circumference, central fat, and also with Avignon's insulin sensitivity index (SiM), fasting insulin, fasting glucose, triglycerides, and systolic blood pressure. rs1885177A/C was only associated with SiM. A protective haplotype was associated with lower SiM, triglycerides, and systolic blood pressure [63]. In a similar study rs914458C/G showed moderate association with T2D [64]. Multiple consistent associations were observed between SNPs rs941798C/G and rs2426159A/G and metabolic parameters reflecting insulin sensitivity and the lipid profile [64], thereby suggesting that *PTPN1* may influence susceptibility to the metabolic syndrome in a French population [63, 64]. The same set of 23 SNPs used by Bento et al. [61] showed an association with measures of atherosclerosis, adjusted for age, sex, and smoking status in Caucasian subjects with T2D [65]. SNPs rs803742C/T, rs2206656C/G, rs16989673ins-/G, and rs914458C/G were used in the statistical analysis, although they did not respect Hardy-Weinberg equilibrium in this cohort [65]. The extensive linkage block was confirmed in several other replication studies, but only weaker or not significant associations with the phenotype could be found. Indeed, Florez et al. [66] failed to detect an association of any SNP or common haplotype with T2D, fasting plasma glucose, and insulin sensitivity in a large collection of Northern European subjects [66]. In Pima Indians, only three SNPs upstream *PTPN1* were nominally associated with a measure of insulin sensitivity in nondiabetic subjects (rs1967439A/G, rs4811074C/T, and rs4811075A/G), and none of them, either singularly or collectively as haplotypes, were associated with T2D [67]. Neither the frequency of the polymorphisms rs3787345C/T, rs754118C/T, rs2282147A/G, rs718050A/G, and rs3787348G/T, nor their haplotypes, differed significantly between cases and control subjects of Polish origin [68].

Subjects carrying a common insertion of a guanosine at position g.-1484, in the 3′UTR, rs16989673ins-/G, showed PTP1B mRNA overexpression in skeletal muscle, and PTP1B mRNA stability was significantly higher in HEK293 cell lines transfected with rs16989673insG, as compared with those transfected with wild-type PTP1B [69]. This variant was associated with features of the metabolic syndrome: higher HOMA-IR index, triglycerides, and total/HDL cholesterol ratio in males, higher blood pressure among females in two Italian cohorts [69]. In Iranian non diabetic subjects, male carriers of rs16989673insG showed significantly higher fasting insulin, total and LDL cholesterol, apolipoprotein B, and HOMA-IR, while in females the BMI only was significantly increased in rs16989673insG carriers [70]. No association with T2D was reported in this study [70], in Pima Indians [67] or in Asian Indians [71]. A large Swedish study failed to replicate the first Italian association [72]. The rs16989673insG was also not associated with BMI [73], fasting glucose, fasting insulin, or T2D in Danes [73] and Scandinavians [66], or with insulin resistance assessed by HOMA-IR or QUICKY index in a small Polish study [74], or with essential hypertension in Caucasian Australians [75]. Interestingly, the previously reported risk allele, rs16989673insG, was significantly associated with lower triglycerides in European women [63], and a study performed on large Hispanic-American families with a low incidence of T2D revealed a significant association with improved insulin sensitivity index (Si), lower fasting glucose, and higher acute insulin response. Data presented in this latest study are however hard to interpret, and in fact only fasting glucose shows a clear pattern of association with the SNP [62]. Consistent with this, Bento et al. [61] reported that the haplotypes containing rs16989673insG had a neutral or even protective effect on the risk of developing T2D [61]. Finally, rs16989673ins-/G was associated with measures of atherosclerosis, adjusted for age, sex, and smoking status in Caucasian subjects with T2D, but caution should be taken in the interpretation of these results, since the distribution of the genotypes at rs16989673ins-/G did not attend Hardy-Weinberg equilibrium expectations [65]. Indeed, the lack of consistency in the associations reported for this SNP could be due to its low minor allele frequency.

A rare nonsynonymous variant, rs16995309C/T (P387L), was reportedly associated with T2D in a Danish sample [73] but not in a small Chinese case-control study [76], a small Finnish sample [77], in Asian Indians [71], or in Germans [78], although P387L showed higher triglyceride levels both in diabetics and in controls [78]. It was also not associated with T2D or BMI in a study performed on Pima Indians [67]. P387L was associated with lower fasting insulin level and glucose disappearance index in two independent cohorts (of Caucasian and African origin) [79], but it was not associated with glucose/insulin parameters or BMI in a study on obese French subjects [80].

No associations were found with T2D for the variants rs2230605A/G R199R in Northern Europeans [66, 73], g.-104ins-/G, g.-86T/G, rs145883911C/T (T420M), IVS9+57C/T, and IVS9+58A/G in Danish [73], and rs2230604C/T (P303P) in several European populations and Asian Indians [66, 71, 73, 80]. Although rs2230604C/T was shown to interact with Avignon's insulin sensitivity index in one study [63], this association was not confirmed [64]. rs2230604C/T was further associated with BMI, waist circumference, triglycerides, and LDL levels but not with blood pressure, glucose, insulin, or leptin levels in Chinese children [81]. The common polymorphism rs16995304A/G (G381S) was not associated with T2D in Northern Europeans and in Pima Indians [66, 67, 73], but it was, however, associated with BMI in this latest ethnicity [67].

The silent variant g.981C/T in exon 8 showed a significant association with the risk of being affected with either IGT or T2D in a Canadian population [82], but it was not associated with several features of insulin resistance in two independent Italian cohorts [69].

IVS6+82A/G was at first associated with hypertension, albuminuria, and HbA1c. However, after adjustment of the lipid and lipoprotein values for the effect of BMI, only the significant association with albuminuria was maintained [77]. In addition, significant associations were observed between the IVS6+82A/G polymorphism and waist circumference, total cholesterol, and LDL-cholesterol levels in Chinese hypertensive patients [83], but no association between IVS6+82A/G and blood pressure, glucose, insulin, or leptin levels were observed in Chinese children [81]. Caucasian subjects carrying IVS6+82G/A were further reported to show higher body fat levels and lower acute insulin response and disposition index [79], especially in presence of leptin receptor gene variants [77].

A small Iranian study evaluated the specific contribution of seven polymorphisms found in the 2 Kb at the 3′ extension of *PTPN1* (plausibly, the promoter region) to the development of T2D [84]. Only rs6126029A/C (g.-1023) showed nominal association with T2D, but this association was not confirmed after correction for established T2D risk factors [84]. Functional analyses in HepG2 cell lines also showed that rs6126029A/C did not influence PTPN1 expression [84]. The IVS5+3666del-/T SNP was only found in one study, and it was associated with morbid obesity in a French cohort, with no effects on T2D development or on glucose/insulin parameters [80].

## 5. INPPL1

INPPL1 (also known as SH2 domain-containing inositol polyphosphate 5-phosphatase-2, SHIP-2) was identified as a 5-lipid phosphatase responsible for the regulation of insulin-signaling by hydrolyzing PI3-kinase products PtdIns(3,4,5)P3 (PIP_3_) to PtdIns(3,4)P_2_ [6]. Overexpression of SHIP-2 inhibits insulin-induced glucose uptake and glycogen synthesis in 3T3-L1 adipocytes and L6 myotubes [85–87]. Genetic ablation of *INPPL1* in mice has generated conflicting results. *Inppl1* knockout mice were originally reported to show lethal neonatal hypoglycemia resulting from insulin hypersensitivity [88], but the same authors later reported that in their original model, in addition to inactivating the *Inppl1* gene, the *Phox2a* gene was also inadvertently deleted [89]. It thus remains to be clarified whether the phenotype of this *Inppl1* ^−/−^ mouse is a consequence of *Inppl1* or *Phox2a* deletion and/or of the inactivation of both these genes. Another *Inppl1* knockout mouse has subsequently been generated, in which exclusively the *Inppl1* gene is inactivated [90]. These animals show normal insulin and glucose tolerance but are highly resistant to weight gain on a high fat diet, exhibiting no increase in serum lipids, insulin, or glucose levels and enhanced insulin-signaling under obesity inducing conditions [90]. 

The human *INPPL1* gene is located in the chromosome 11q13-14, region that has been suggested to be linked to T2D with insulin resistance and hypertension [91–93]. A first study identified in a small cohort of eight T2D subjects, a 16 bp deletion in the 3′-untranslated region of the *INPPL1* gene (Figure 2). The deleted region included a potential conserved sequence element thought to be important for regulation of mRNA stability and translation efficiency, and indeed this deletion was shown *in vitro* to increase *INPPL1* expression levels. When the frequency of the mutated allele was assessed in a population of 415 diabetic subjects from the United Kingdom and Belgium, compared to 567 healthy controls, 9 subjects carrying the mutant form were found in the diabetic cohort, versus 3 in the control group [94]. 

In a more recent study, the same authors carried out an extensive resequencing of the *INPPL1* gene (15.2 Kb), including all exons and introns, in a cohort of 64 individuals [95]. A total of 49 variants were initially described, but only 11 markers (rs77348083A/C, rs144989913-/GCTCCTTGCGGGCTGGCGTGGACCGGGA, rs12288631C/T, rs2276048A/C/G/T, rs2276047C/T, rs12361913C/T, rs1006488C/T, rs61736312A/G, rs10751199A/G, rs11235472C/G, and rs9886C/G) and the previously identified 16 bp deletion in the 3′ untranslated region were further analyzed, since several of the initially observed mutations were identified as rare variants, and SNPs rs77348083A/C, rs74635729A/G, rs79054886A/G, and rs76870980G/T showed a complete linkage disequilibrium (Figure 2). The selected markers represent 79% of haplotype variation in the gene. They were genotyped in 1,304 individuals from 424 British T2D families from the Diabetes in family (DIF) study collection and were confirmed to be in Hardy-Weinberg equilibrium. The strongest evidence for association in this collection was between hypertension and a group of three SNPs, rs2276047, rs144989913, and rs9886, which were also associated with central obesity [95]. Furthermore, rs2276047 and rs144989913 together showed evidence for association with T2D (*P* = 6.2 × 10^−4^) and with metabolic syndrome, while rs2276047 and rs9886 together were associated with general obesity (*P* = 1.5 × 10^−3^), and the most common haplotype (rs144989913, rs2276047, and rs9886) had the single best haplotype association with all five traits examined. Finally, the 16-bp deletion in the 3-untranslated region of *INPPL1 *was not associated with any of the phenotypes measured in the DIF cohort, even if it occurred in 2.0% of the diabetic patients and in only 0.7% of control individuals. Kaisaki et al. were, however, unable to confirm these findings in an independent cohort of 905-unrelated French type 2 diabetic patients and control subjects; in this cohort they observed an association between the insertion variant of rs144989913 and hypertension [95]. A subsequent study in hypertensive subjects without diabetes or metabolic syndrome did not confirm this association suggesting that *INPPL1 *variants may be specifically involved in mechanisms causing hypertension in insulin-resistant patients [96]. Genotyping of a cohort of 106 Japanese type 2 diabetic and 100 nondiabetic control subjects identified 10 additional SNPs including 4 missense mutations [97]; one of these SNPs, a leucine to isoleucine substitution at position 632 (rs61749195A/C) was observed with increased frequency in nondiabetic subjects, suggesting that this mutation might exert a protective action toward insulin resistance. This hypothesis was sustained by transfection studies showing that expression of rs61749195A-*INPPL1* inhibited insulin-induced PIP_3_ production and Akt phosphorylation less potently than the wild-type *INPPL1* in CHO-IR cells.

## 6. TRIB3

The pseudokinase TRIB3 binds Akt, inhibiting downstream insulin-signaling [7]. It has been shown, in cellular and animal models, that changes in TRIB3 expression levels induce systemic insulin resistance [7, 98, 99]. A *TRIB3* missense SNP (i.e., Q84R, where arginine (R) replaces glutamine (Q) at position 84; rs2295490A/G) has been described [100] (Figure 2), with a global minor allele frequency of 14.4%, varying from 13% in European and African subjects to 25–27% in Japanese and Chinese subjects. Several evidence from *in vitro* studies suggest that this amino acid change acts as a gain-of-function substitution. In fact, HepG2 hepatoma cells overexpressing the TRIB3 R84 variant show a greater reduction of insulin-stimulated Akt phosphorylation than those expressing similar amounts of the Q84 TRIB3 form [100]. Likewise, transfection of TRIB3 R84 into dispersed human islet cells, as well as into rat MIN6 beta cells, results in a stronger inhibitory effect on Akt activation, that was paralleled by an impaired glucose-stimulated insulin secretion [101]. Importantly, similar data have also been reported in human vein endothelial cells (HUVECs) naturally carrying the *TRIB3* Q84 or R84 variant [102]; in these primary cell line, insulin-stimulated Akt phosphorylation was significantly reduced in the presence of R84 TRIB3 form. In addition, R84 carrying cells also showed a blunted response to insulin in terms of eNOS activation and nitric oxide (NO) release, two important Akt-mediated endothelium specific actions of the hormone. These data are supported by the results of an *in silico* bioinformatic analysis showing that the Q to R amino acid change at position 84 alters intramolecular salt bridge formation, thus making TRIB3 R84 a stronger Akt binder and inhibitor than TRIB3 Q84 [102]. *In vivo* rs2295490 has been associated with insulin resistance [100], defective insulin secretion [101, 103, 104], T2D [100], and CVD [100]. In a first study, Prudente et al. showed that rs2295490 was significantly (*P* < 0.05) associated with several insulin resistance-related abnormalities in two independent cohorts (*n* = 178 and *n* = 605) of nondiabetic Italian subjects and with increased cardiovascular risk in 716 T2D patients (OR 3.1 [95% CI 1.2–8.2], *P* = 0.02); furthermore in a separate cohort of 100 T2D individuals who survived myocardial infarction, age at MI was progressively lower in homozygous (RR) and heterozygous (QR) carriers of the R84 variant than among QQ carriers [100]. In a subsequent study on 645 nondiabetic individuals from Italy, early insulin secretion adjusted for the level of insulin resistance (i.e., the disposition index (DI)) was shown to be significantly reduced in individuals carrying the R84 variant compared with homozygous QQ carriers [103]. Similar data were obtained by a second study on an independent sample of 791 individuals from Italy [104]; when the two studies were meta-analyzed, a 25% and 50% reduction of the DI was reported in QR and RR subjects, respectively. The association of rs2295490 with impaired insulin secretion was described also in a Polish cohort of 766 patients with T2D; RR homozygous individuals from this cohort exhibited 30% lower plasma C peptide levels than QQ subjects [101]. Prudente et al. carried out, also, a case-control study for impaired glucose regulation (IGR, i.e., either T2D or impaired fasting glucose and/or impaired glucose tolerance) on a cohort comprising a total of 6634 individuals of European ancestry, recruited in Italy and in the United States as a part of the GENIUS (genetics of type 2 diabetes in Italy and United States) consortium efforts [104]. They report that *TRIB3 *R84 variant is significantly associated with IGR, with an overall OR for the additive model of inheritance (i.e., risk increase for each copy of the R84 variant) of 1.19 (95% CI 1.06–1.34). Interestingly, most of the observed association was due to association with IGR diagnosed before age 45 years (early IGR) (OR 1.31 *P* < 0.00007) [104]. This observation suggests that, similarly to what has been observed for *ENPP1* rs1044498 SNP, more than affecting the overall risk of abnormal glucose homeostasis, the rs2295490 *TRIB3* may anticipate its appearance in predisposed individuals. 

Unfortunately, it has been so far impossible to confirm or disprove the findings from classical association studies with GWAS data since neither rs2295490 nor any good proxy of this variant has been included in GW association studies [33].

## 7. Conclusions

In this Review, we have summarized the available evidence on the role of polymorphisms in the genes encoding for insulin-signaling inhibitors molecules in determining genetic predisposition to T2D and related diseases. Overall, solid evidence seems to exist only for rs1044498 of the *ENPP1* gene and for rs2295490 of the *TRIB3* gene, whose association with T2D risk and insulin resistance, even if not confirmed (for *ENPP1*) [33] or not yet investigated (for *TRIB3*) [33] by GWAS studies, has been consistently reported by several original studies [16–20, 22–33, 38–43, 100, 101, 103, 104] and large meta-analyses [32, 104]. It is worth underlining that both rs1044498 and rs2295490 have been reported to be associated not only with defective insulin action in peripheral target tissues but also with impaired insulin secretion and decreased beta-cell homeostasis [14, 15, 101, 103, 104]. These observations suggest that the two major pathogenic defects of T2D share common genetic causes and support the hypothesis that they should be seen as different aspects of the same process rather than as separate events [105]. In addition, several studies have shown that the effect of rs1044498 and rs2295490 is more evident on early-onset T2D [26, 28, 104]; notably similar data have been obtained for rs1801278 of *IRS1* gene [106]; these data hint to the possibility that focusing on early-onset cases may represent a successful strategy to study the contribution of insulin-signaling gene variants to T2D pathogenesis. Interestingly, a very recent study [107] has investigated the combined role of rs1044498 of the *ENPP1* gene and for rs2295490 of the *TRIB3* gene together with rs1801278 of *IRS1* gene, on CVD, age at MI, and *in vivo* insulin sensitivity reporting a significant additive effect among the risk variants; notably the joint predictive power of ENPP1 rs1044498, IRS1 rs1801278, and *TRIB3* rs 2295490 SNPs was even more evident among obese individuals [107]. These results not only further reinforce the importance of rs1044498 and rs2295490 in determining the risk of insulin resistance and related diseases but further underlie that in any single individual the effect of each specific variant is also significantly influenced by the interaction with other variants as well as by environmental factors [108, 109]. Indeed T2D, CVD, IR, obesity, and related metabolic disorders are characterized by extremely heterogeneous phenotypes; thus some of the earlier positive findings reported in this Review that were not confirmed in subsequent, larger studies may have been “real” associations, even if limited to a specific subset of subjects in a definite environmental and genetic setting. In fact the extreme hetereogeneity of T2D and related diseases may represent one of the main reasons for the apparent discrepancy between the results of GWAS and those of classical “candidate-gene” studies, as the design of GWAS does not take into account several factors, including sexual dimorphism, age at disease onset, and obesity status, that have been shown to have an important role in the pathogenesis of metabolic diseases. In recent years, several methods for screening gene-environment interaction have been proposed [110] and their wider implementation is likely to shed further light on the genetics of metabolic diseases. Furthermore, novel technologies, such as next generation sequencing, that allow to address the role of relatively rare variants, will significantly contribute to obtain a clearer picture of the genetics basis of T2D and related diseases [111]. Finally, the data on the genetics of insulin-signaling inhibitors molecules, recapitulated in this Review article, may supply useful elements to interpret the results of novel, more technically advanced, genetic studies; indeed it is becoming increasingly evident that genetic information on complex metabolic diseases should be interpreted taking into account the composite biological pathways underlying their pathogenesis [112]. In addition, as suggested by recent studies on *ENPP1* rs1044498 [35–37], a deeper knowledge of the genetic variants affecting the pathogenesis of T2D and related metabolic diseases may have important implications also for the implementation of tailored therapeutical approaches.
